# Driving older adults’ online engagement through an integrated service experience: a mixed-methods study in an online–merge-offline educational context

**DOI:** 10.3389/fpubh.2026.1695630

**Published:** 2026-03-17

**Authors:** Yi Mei, Ze Wang

**Affiliations:** School of Finance, Zhejiang University of Finance & Economics, Hangzhou, Zhejiang, China

**Keywords:** digital literacy, educational platforms, integrated service experience, older adults’ online engagement, online-merge-offline

## Abstract

**Objective:**

This study examines how integrated service experience shapes older adults’ online engagement in online-merge-offline (OMO) platforms and investigates the role of digital literacy in this process.

**Methods:**

Using a mixed-methods design, we built a theoretical framework through grounded theory based on interviews and secondary data. We then applied Partial Least Squares Structural Equation Modeling (PLS-SEM) to test the framework using survey data.

**Results:**

Integrated service experience, which consists of offline service experience and online–offline integration, significantly increases older adults’ engagement. Offline service experience improves satisfaction with offline services, and both this satisfaction and online–offline integration heighten perceived value of online services. Higher perceived value, in turn, promotes online engagement. Digital literacy directly increases engagement and strengthens the effect of perceived value on online engagement.

**Discussion:**

These findings clarify the mechanisms of older adults’ online engagement in OMO educational platforms and extend lifelong learning and geragogy theories into digitally blended environments. They also offer practical guidance for designing age-friendly and motivation-sensitive OMO strategies that support inclusive and sustained later-life learning.

## 1 Introduction

Amid rapid global population aging, governments are advancing policies that emphasize lifelong learning, digital well-being, and social connectedness as key elements of later-life development (1, 2). In China, active aging has become a national priority, and the “Internet Plus Older Adult Education” initiative has accelerated the development of Online Merge Offline (OMO) learning models across education, technology, and public health sectors (3). OMO platforms combine structured online courses with organized offline activities, supporting continuous learning, digital psychological welfare, and peer interaction (101, 103). Evidence shows that these platforms enhance practical skills, strengthen social engagement, and expand access to learning, improving older adults’ quality of life (4).

Compared with younger learners, whose engagement is mainly skill-driven (5, 6), older adults value social integration and emotional connection in collective learning settings (7, 8, 105). Their engagement depends heavily on community size, which supports resource sharing and easier peer matching (9–11). Because OMO platforms blend offline instruction with flexible online access, the online component plays a key role in quickly attracting and sustaining large learning communities.

Most studies of online engagement focus on younger users (12–14), so we still know little about how older adults engage. Younger learners usually join fully online courses on their own to gain specific skills, driven by platform features. Older adults, however, typically start in offline settings such as community universities or study groups and then move online to continue learning and maintain real-life relationships (15–17). This offline-led and online-extended pattern highlights the need for further research on older adults’ engagement mechanisms.

Many older adults face significant variation in digital literacy, unlike younger “digital native” cohorts. Within the older population, some rely only on basic phone functions, while others struggle with technology anxiety, cognitive or physical limitations, and limited support (18–20). Many also show low motivation to develop new digital skills (21). These intra-generational disparities create persistent digital divides and shape how older adults access online services and how they engage once they enter digital learning environments.

This study therefore poses the following research questions:

*Research Question 1*: How do integrated service experiences influence older adults’ online engagement with OMO educational platforms?

*Research Question 2*: How do digital literacy disparities among older adults affect their online engagement with OMO educational platforms?

Using grounded theory, we build a framework explaining how offline service experiences shape older adults’ online participation in OMO platforms and validate it with survey data. The findings clarify key engagement processes and show how well-designed services can enhance digital well-being, reduce participation barriers, and support active aging. They also offer practical guidance for creating OMO systems that enable more inclusive and sustained digital engagement among older adults.

## 2 Literature review

### 2.1 Older adults and OMO educational platforms

The United Nations defines people aged 65 and above as older adults based on the retirement age in Western developed countries (22). The World Health Organization defines the start of old age as 55 years old in combination with the retirement age in developing countries, and points out that the characteristics of older adults lie in the loss of previous social roles or the inability to make positive contributions to society (23). Scholars generally regard retirement as a marker of entry into old age because it signifies a major shift in an individual’s social role. In some developing countries, the retirement age is set at 50 for certain groups, and scholars such as Burmeister (24) therefore define people aged 50 and above as older adults. Following this approach, this study uses retirement as the key criterion for identifying older adulthood and defines older adults as individuals aged 50 and above.

Scholars such as Blazer and Dan (25) and Hussenoeder et al. (26) have highlighted that older adults may experience age-related changes, including physiological adjustments and psychosocial transitions, following retirement. In response, researchers like Boulton-Lewis et al. (27), Walker (28), Fries (29), and Foster and Walker (30) emphasize the importance of promoting mental well-being and social reintegration among older adults through meaningful collective learning and integrated support systems, in alignment with the goals of active aging. Notably, digital platforms now offer new possibilities through OMO educational platforms—which facilitate group-based learning around shared interests such as calligraphy and tai chi. These platforms effectively connect older adults with common educational and recreational pursuits, fostering interest-driven collective learning and resource sharing within a digitally enhanced environment (3, 4, 31). Current studies mainly examine how technology features (32, 102), personal traits (33, 34), and motivational factors (35, 36) affect digital engagement, but the discussion on the platform’s offline service influencing older adults’ digital engagement, particularly in older adulthood OMO educational platforms is not sufficiently in-depth (37). This gap is paradoxical considering the hybrid nature of most OMO educational platforms.

### 2.2 Older adults’ engagement in online-merge-offline educational contexts

Given older adults’ established reliance on offline service, eldely educational platforms typically adopt an OMO model to encourage older adulthood engagement with online service, such as interacting with and providing support to providers and their fellows online. OMO model originates from O2O model (38). However, compared with the latter, it emphasizes the deep integration of online and offline service more strongly. Central to this model is leveraging internet technologies to integrate offline resources and achieve seamless transitions between physical and digital services. For instance, OMO educational platforms for older adults may use online platforms to distribute activity information and allow course bookings while organizing corresponding offline events and classes, enabling older adults to engage in social and recreational activities within familiar environments.

Existing literature predominantly focuses on familial support (e.g., children assisting with technology) or older adults’ intrinsic motivation (e.g., information seeking) (15, 39), while insufficient attention has been paid to service interactions for promoting engagement in OMO educational platforms for older adults. Under OMO models, the deep integration of online-offline service allows offline service experiences to establish an accessible and credible foundation for older adults’ understanding of online value (40). Service interactions formed during offline engagements – including interactions with service providers and between customers – play pivotal roles in advancing OMO models within OMO educational platform for older adults. Offline service uniquely drive online engagement by serving as a bridge for interest activation. For example, platform-organized offline activities such as square dancing and tai chi sessions not only initiate engagement but also effectively stimulate online exchanges of learning experiences among older adults, whereas purely online approaches often fail to achieve such organic interaction (41). Additionally, peer-to-peer interaction during the activities organized by OMO educational platforms will motivate older adults engagement to relationship reinforcement (42). Under the OMO model, the integration of online and offline service is reflected in the fact that online service are the reinforcement and deepening of offline interactions (43, 44). For example, offline service are restricted by time and location, while online service can further carry out personalized interactions and connections at any time and place (45).

When navigating online service, older adults face significant learning costs in information seeking, exchange, evaluation, and utilization (39, 46, 47). Digital literacy critically determines these costs, defined as the capacity to use digital tools to search, evaluate, and apply information for personal needs, while managing technological risks and verifying information authenticity (48, 49). There are pronounced intra-group disparities in the digital literacy of older adults (15). Digitally proficient older adults can adapt reaily, while disadvantaged cohorts show severe limitations. Nearly half of them avoid information searches because of technical deficits, insecurities in experience evaluation, and a resistance to skill development without extrinsic motivation (21, 50). For this latter group, online service value perception hinges overwhelmingly on offline-derived firsthand experiences. Under OMO models, integrated physical-digital services bridge comprehension gaps by leveraging tangible interactions (46, 51), yet persistent literacy gaps amplify operational barriers during online community engagement (97, 100, 101).

## 3 Qualitative analysis

### 3.1 Research procedures and data collection

The study employed in-depth interviews as the primary data three groups, namely collection method, rigorously examining how offline service drive older adults’ online engagement. The research selected older adults and service providers from three distinct learning platforms, namely the Hangzhou Financial Literacy Program, the Hangzhou Square Dancing Learning Community, and the Wuhu Tai Chi Learning Group, as the study subjects. These OMO educational platforms align with the common hobbies of older adults (15, 52). Their operations are relatively stable, possessing a certain degree of representativeness and reference value. Moreover, the researchers have established good cooperative relationships with the platform organizers and service providers, facilitating multi-role research. Researchers conducted 20 individual interviews (≥40 min each) with service providers and older adults from three OMO educational platforms: supplemented by 6 focus group interviews (≥60 min each) using protocols detailed in Table 1. Secondary data from policy documents, academic literature, news reports, official websites, and industry analyses (Table 2) were integrated, compiling over 100,000 words of textual material. Throughout theoretical sampling, iterative data collection and three-stage coding (open-axial-selective) deepened conceptual understanding until achieving theoretical saturation. Triangulated coding by three researchers ensured objectivity and scientific rigor.

**Table 1:** Interview questions for older adults and service providers.

Interviewee	Interview questions
Older adults	Which experiences during *offline service* left the deepest impression on you? What feelings arose during offline service? *Specifically explain why these feelings emerged.* Which attributes of *online service* do you value most? After experiencing offline service, what expectations or perceptions do you have regarding online service? *Please recall in detail:* What subsequent behavioral intentions toward online service did you develop after offline service experiences? *Elaborate on the reasons for these intentions.*
Service providers	To drive online service adoption through offline experiences, what kind of offline experiences would you design for customers? How do you define the relationship between offline and online service?

**Table 2:** Overview of primary and secondary data sources.

Type	Source	Description
Primary data	Individual in-depth interviews	20 interviews with service providers and older adults via telephone, WeChat, and face-to-face communication (each exceeding 40 min)
	Focus group interviews	6 group discussions with multiple service providers and older adults (60 min each)
	Participant observation	10 on-site inspections at offline service locations
Secondary data	Internal documents	20 internal reports and business plans related to OMO educational platforms
	Industry research reports	10 industry analysis reports
	Media coverage	15 media reports

### 3.2 Grounded theory analysis

Grounded theory (53) is a systematic methodology for collecting and analyzing data to develop scientific theories reflecting phenomena’s essence. This study adopts grounded theory for three reasons: First, it aligns with our aim to explore how offline service experiences influence elderly online behavioral intentions under OMO models. Given limited literature, grounded theory enables conceptual clarification and theoretical framework construction. Second, the research problem’s complexity, absence of quantitative databases, and lack of mature theoretical frameworks make large-scale surveys ineffective. Grounded theory overcomes these limitations through qualitative depth. Third, the methodology synthesizes multi-source data (interviews, WeChat/email records, literature, news, industry reports, etc.) to extract theoretical insights from extensive datasets.

#### 3.2.1 Open coding

Open coding systematically transforms raw data into core concepts, analyzes their interconnections, and synthesizes them into higher-order categories (54). This study initially generated 103 concepts, which, through further analysis and classification (including removal of invalid concepts and merging of similar ones), were ultimately consolidated into 9 categories. Each category is exemplified by 1–2 raw statements to illustrate its formation (Table 3).

**Table 3:** Open coding results with sample statements and categories.

Raw statement example	Conceptualization	Categorization
“The teacher teaches very conscientiously, answers questions promptly, and instructs me repeatedly. (c-3-200705)”	Interaction experience between older adults and teachers	Interaction quality
“Other older adults are also very friendly, and we get along pleasantly. (c-2-200808)”	Interaction experience among older adults	Peer-to-peer interaction quality
“I’m very satisfied with the teacher’s teaching content and attitude. (c-3-200704)”	Satisfaction with service providers	Satisfaction with offline service
“The service organizer this time is worthy of praise. (c-1-200614)”	Satisfaction with service institution	
“I think there are no flaws in my interactions with others. (c-2-200808)”	Satisfaction with other customers	
“Overall, I’m very satisfied with the entire service experience. (c-1-200613)”	Overall service satisfaction	
“I can study math now while sharing related videos and graphics in the interest-based learning community. (c-1-200612)”	Online-offline resource sharing	
“All other teachers in our interest-based community collaborate with us, ensuring seamless coordination. (s-3-200803)”	Online-offline collaboration	
“I can quickly upload my learning experiences to the online platforms. (c-1-200620)”	Rapid content contribution	
“I can promptly access the online forum to discuss offline gatherings. (c-2-200809)”	Real-time peer interaction	
“When free, I watch instructional videos in the platfoms and give likes, which makes me happy. (c-1-200621)”	Positive psychological feelings	Emotional value
“I use the online platforms to communicate with mentors and peers. (c-1-200620)”	Beneficial social connections	Social value
“Information in the online platforms matches my needs. (c-3-200712)”	Useful information	Information value
“I use WeChat; I wonder if communication here conflicts with it. (c-2-200816)”	Multi-platform usage concern	Digital literacy
“I worry complex navigation might prevent me from finding my favorite mentor’s content. (c-3-200718)”	Information accessibility concern	
“Frequently used content is on the homepage for easy access. (c-1-200619)”	Efficient resource retrieval	
“I’m concerned about potential ads—I’ve experienced this with other platforms. (c-2-200822)”	Content trustworthiness evaluation	
“I focus solely on target information in the community; irrelevant content is ignored. (c-1-200621)”	Selective information consumption	
“Learning this platform interface might take effort. (c-3-200719)”	Platform learning curve	
“The interface should not be hard to learn—the font is large and very intuitive. (c-1-200613)”	Customer-friendly design perception	
“I expect to use this interest hub frequently. (c-2-200823)”	Online platform use intention	Engagement with online service
“After offline activities, I share experiences online, view others’ posts, give likes, and rewrite mentors’ key takeaways repeatedly. (c-1-200620)”	Online-activity participation	

c/s-1/2/3- ****** is the coding for the type of interviewees, the interview location, and the interview time. Among them, represents that the interviewee is an older adult, and represents that the interviewee is a service providers; 1 refers to the offline service point of interest-based community 1, 2 refers to the offline service point of interest-based community 2, 3 refers to the offline service point of interest-based community 3; ****** is the digital coding of the interview time. The first two digits represent the year (i.e., 20**), the middle two digits represent the month, and the last two digits represent the specific date.

#### 3.2.2 Axial coding

During the axial coding process, three scholars and practitioners in care services for older adults were first invited to screen all categories and measurement items. Subsequently, the research team conducted comparative discussions on the screened results to identify core categories and their corresponding sub-categories. Logical relationships among the categories were systematically mapped, resulting in six core categories: offline service experience, inherent states of offline service, online service attributes, perceived value of online service, characteristics of older adults, and online behavioral intentions of older adults (Table 4).

**Table 4:** Axial coding structure with core categories and definitions.

Core category	Corresponding sub-category	Category definition
Offline service experience	Interaction quality	Older adults’ evaluation of service delivery methods during interactions with service providers
	Peer-to-peer interaction quality	Perception of interactions with other customers during service experiences
Inherent states of offline service	Satisfaction with offline service	Positive evaluation of service providers, affiliated institutions, other customers, and overall service process
Online service attributes	Integration of online-offline service	High relevance between community platform services and offline service content, personnel, and information
Perceived value of online service	Emotional value	Ability to spend enjoyable time through community platform engagement
	Social value	Ability to fulfill social interaction needs through community platform activities
	Information value	Ability to obtain needed or relevant information through community platform services
Older adults’ characteristics	Digital literacy	Ability to use internet and digital tools to communicate, search, and process information for personal needs
Online behavioral intentions	Engagement with online service	Willingness to use community platforms for services, social interactions, and activities

#### 3.2.3 Selective coding

**Figure 1:**
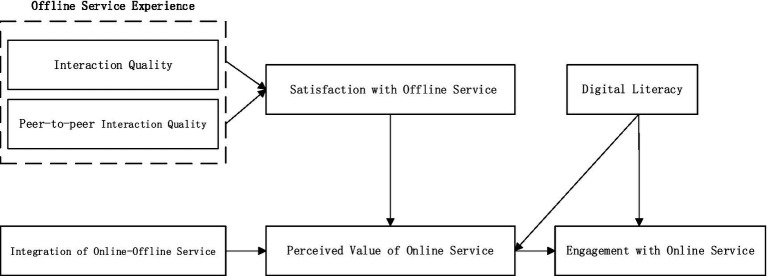
Mechanism linking offline service to online engagement in OMO contexts.

Selective coding involves selecting the core category based on the main categories. Specifically, it further analyzes the main categories to generate a core category that can encompass all the main categories, thereby forming a story line with a complete explanatory framework. This study utilizes the “causal relationship” coding method to sort out and determine the core category and construct a theoretical model. Through grounded theory analysis, after the coding is completed, no remaining categories are found, indicating good theoretical saturation. Finally, all the main categories point to the core category of “the mechanism of offline service driving older adults to engage with online service under OMO educational contexts” (Figure 1). The positive offline experiences of older adults result in their satisfaction with offline service. The satisfaction of older adults with offline service and the integration degree of offline and online service positively influences the perceived value of older adults for the online service, which in turn affects their online engagement. Digital literacy has a positive impact on their online engagement and further moderates the relationship between its perceived value of online service and online engagement.

This study derived core categories through grounded theory methodology and established causal relationships among them to construct a preliminary theoretical model. The model identifies influencing factors of older adults’ engagement with online service in offline-driven contexts. However, conceptual definitions of some influencing factors remain at the qualitative research level. Furthermore, the interrelationships among these factors exhibit significant variations, necessitating further validation of the theoretical model through quantitative analysis methods.

## 4 Theoretical framework and hypothesis development

### 4.1 Research model

**Figure 2:**
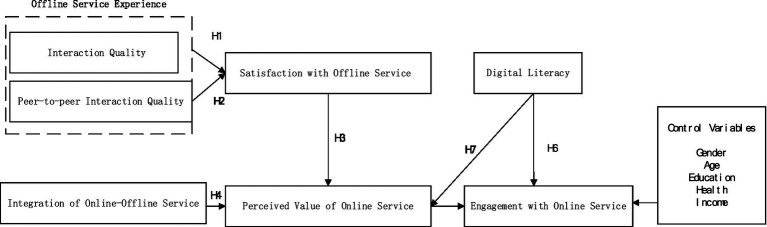
The proposed model with hypothesis.

According to the results of previous interview studies and grounded theory qualitative analysis, a theoretical framework on the mechanism of the role of offline service in driving older adults to engage with online service under the OMO model was formed. In addition, academic consensus remains limited regarding how demographic variables influence older adults’ behaviors. Consequently, this study adopts demographic controls commonly employed in gerontological research (15), including sex, age, education level, health status, and income. The finalized theoretical model is presented in Figure 2.

### 4.2 Hypothesis development

#### 4.2.1 Interaction quality and satisfaction with offline service

Interaction quality refers to the customer’s evaluation of the service delivery during interactions with the provider. This factor is a critical antecedent to satisfaction (106, 107). Theoretically, service providers’ behaviors, such as professionalism, warmth, and responsiveness, shape the overall service experience (55, 56). In the context of OMO educational platforms for older adults, high-quality interactions lead to positive psychological outcomes, including affective benefits (e.g., enjoyment) and cognitive benefits (e.g., acquiring new skills) (54, 57). Empirical evidence from participant testimonials further supports this, with praise for “meticulous guidance” and “individualized coaching” during structured activities. Therefore, the following hypothesis is proposed (96, 98).

*H1*: Interaction quality positively influences older adults’ satisfaction with offline service.

#### 4.2.2 Peer-to-peer interaction quality and offline service

Beyond provider-customer interaction, the behavior of other customers also affects the service experience (58). Verhoef et al. (108) and Lemke et al. (59) highlight the importance of peer interactions in shaping the overall customer experience. In offline interest activities for older adults, peer interactions such as mutual assistance and friendly behavior enhance the service experience, promoting satisfaction. This is confirmed by interview data showing that older adults value the social support and camaraderie found during interactions (60, 61). Thereby the following hypothesis is proposed:

*H2*: Peer-to-peer interaction quality is positively correlated with older adults’ satisfaction with offline service.

#### 4.2.3 Satisfaction with offline service and perceived value of online service

Perceived value refers to customers’ assessment of a service’s ability to fulfill intended purposes or execute core functions (62). It includes both post-experience evaluations and pre-engagement expectations (63). In this study, the perceived value of online service refers to older adults’ expectations of an OMO educational platform’s ability to satisfy social, informational, and entertainment needs following positive offline experiences. This expectation is driven by satisfaction with offline service, where positive provider/peer interactions create a transference effect, enhancing confidence in the platform’s value proposition (64). Evidence from field studies shows that satisfactory offline experiences lead to higher perceived value of the associated online service across various OMO platforms. For example, Tai Chi group participants noted that online services’ reliability, mirroring offline instructor quality, was key to their engagement. One participant stated, “Positive offline experiences make me expect even greater value from the online features. Thereby the following hypothesis is proposed:

*H3*: Older adults’ satisfaction with offline service is positively correlated with their perceived value of online service.

#### 4.2.4 Integration of online-offline service and the perceived value of online service

Integration of online-offline service refers to how well online and offline services are coordinated, including staff consistency, content complementarity, and real-time information flow. Studies show that older adults tend to resist digital content disconnected from their offline social networks, as they primarily seek to reinforce real-world relationships through online engagement (65). Higher service integration increases older adults’ perceived value of online services. Field interviews confirm this, with participants highlighting that mismatched content or personnel between channels reduces perceived value. One participant said, “I use the platform to consolidate offline knowledge and communicate with instructors. Unrelated content is not relevant.” Thereby the following hypothesis is proposed:

*H4*: Integration of online-offline service positively influences older adults’ perceived value of online service.

#### 4.2.5 Perceived value of online service and engagement with online service

Perceived value theory asserts that customers’ subjective assessment of a product or service’s utility is crucial in driving engagement (66, 67). Extensive research confirms that perceived value is a key determinant of online service adoption across various technological contexts (45, 68). For older adults using OMO educational platforms, value is derived from enjoyable leisure experiences, fulfilling social interaction needs, and accessing relevant information (69). Therefore, when older adults perceive that an online service aligns with their expectations, they are more likely to engage with and use the service (42). Thereby the following hypothesis is proposed:

*H5*: Perceived value of online service positively influences older adults’ engagement with online service.

#### 4.2.6 Digital literacy and engagement with online service

Digital literacy is the ability to access, evaluate, and use digital information to meet personal needs (70). Research shows significant disparities among older adults, with many exhibiting technophobia and resistance to digital interfaces (46, 71, 72). Engaging with online services can be challenging for those with low digital literacy (99). One participant noted, “I’ll stick to offline activities; mobile interfaces are beyond my skills.” In contrast, those with higher digital literacy navigate technology more easily, reducing fears of abandonment, as one participant said, “Learning this software shouldn’t be hard—large fonts and intuitive design make exploration manageable.” Therefore, digital literacy is crucial in determining older adults’ ability to engage with online services. Thereby the following hypothesis is proposed:

*H6*: Digital literacy positively influences older adults’ engagement with online service.

#### 4.2.7 The moderating roles of digital literacy

Older adults with higher digital literacy are more familiar with digital interfaces, allowing them to master platforms faster and engage more easily (73). When they perceive value, they achieve goals with less effort, increasing engagement (74). In contrast, low-literacy individuals feel the barriers outweigh the benefits (51), with one participant saying, “The complexity diminishes the value.” Higher literacy levels lead to smoother integration, as one participant noted, “Effortless browsing and interactive features match my leisure habits.” This shows the moderating role of digital literacy in engagement. Thereby the following hypothesis is proposed:

*H7*: Digital literacy positively moderates the relationship between perceived value of online service and older adults’ engagement with online service.

## 5 Scale development and data collection

### 5.1 Scales

Based on the citation of authoritative literature, the scale has made adaptive adjustments and innovative developments in combination with the previous qualitative analysis results for the two fields of older adults and OMO educational platforms, which are rarely covered by existing tools, in the research (Table 5). Interaction quality was measured using three items adapted from Choi and Kim (75), peer-to-peer interaction quality was measured using three items adapted from Choi and Kim (75). Satisfaction with offline service was measured using 4 items adapted from Hsieh et al. (76). Perceived Value of Online service was measured using three items adapted from Kim et al. (77). Integration of online-offline service was measured using three items adapted from Yang et al. (64). Digital Literacy was measured using three items adapted from Audrin et al., (70) and Riina et al., (78). Engagement with online service was measured using 2 items adapted from Mei (15).

**Table 5:** Measurements.

Construct	Items	Source
Interaction quality	1. You perceive your interactions with service providers as high-quality.	Choi and Kim (75)
	2. Service providers address your personal needs and preferences attentively.	
	3. Service providers deliver friendly and courteous service.	
Peer-to -peer quality	1. You have positive interaction experiences with other customers.	Choi and Kim (75)
	2. You are confident about maintaining good rapport with other customers.	
	3. Your communication with other customers remains smooth across all stages.	
Satisfaction with offline service	1. You are satisfied with offline service.	Hsieh et al. (76)
	2. You are satisfied with interactions with other customers.	
	3. You are satisfied with service providers.	
	4. Offline service meet your expectations.	
Perceived value of online service	1. You believe online service can fulfill your emotional needs.	Kim et al. (77)
	2. Online service facilitate convenient connections with providers/peers.	
	3. Online service enable access to valuable information.	
Integration of online-offline service	1. Online and offline service content is highly consistent.	Yang et al. (64)
	2. Service personnel are identical or maintain close collaboration.	
	3. Customers/providers can instantly upload offline experiences to online platforms.	
Digital literacy	1. Rate your ability to communicate via internet/social tools.	Audrin et al. (70); Riina et al. (78)
	2. Frequency of requiring assistance with software/internet operations.	
	3. Rate your ability to search information using online tools.	
	4. Rate your ability to analyze, organize, and synthesize digital information from multiple sources.	
Engagement with online service	1. You will use online platforms to interact with staff/other older adults.	Mei (15)
	2. You will engage in various online interactive services.	

### 5.2 Data collection procedure

#### 5.2.1 Pretest

Prior to the formal survey, a pretest was conducted with 15 elderly people to refine survey items. Their feedback guided revisions for linguistic clarity and contextual relevance to older adults. The questionnaire was further validated through double-checking with 100 elderly people recruited via community-based snowball sampling to ensure comprehensibility across diverse literacy levels (79, 80). An exploratory factor analysis (EFA) was performed using SPSS 20 to test the measurement scales (Table 6) which were greater than 0.40. Furthermore, the results present the total variance, showing the eigenvalues of the obtained latent variables. Moreover, the findings provided reliability values based on Cronbach’s alpha (81). Reliability analysis (i.e., Cronbach’s alpha) of the extracted factors was also conducted to ensure that each observed variable had a value greater than 0.70 (82).

**Table 6:** Exploratory factor analysis.

Construct	Indicator	Factor loading	Cronbach’s *α*	Cumulative variance explained
Interaction quality	INT1	0.875	0.786	9.329%
	INT2	0.857		
	INT3	0.519		
Peer-to-peer interaction quality	PTP1	0.805	0.847	19.530%
	PTP2	0.843		
	PTP3	0.681		
Satisfaction with offline service	SAT1	0.726	0.808	32.631%
	SAT2	0.714		
	SAT3	0.801		
	SAT4	0.802		
Integration of online-offline service	IOOS1	0.858	0.761	42.241%
	IOOS1	0.740		
	IOOS3	0.813		
Perceived value of online service	PV1	0.860	0.826	52.988%
	PV2	0.815		
	PV3	0.778		
Digital literacy	DIG1	0.813	0.731	63.927%
	DIG2	0.747		
	DIG3	0.557		
	DIG4	0.771		
Engagement with online service	ENG1	0.851	0.899	71.923%
	ENG2	0.854		

#### 5.2.2 Formal test

We conducted an online survey using a professional survey website called Credamo,^1^ a professional survey platform that ensures data quality through technical controls including required response settings and completion time monitoring (83). To ensure respondents had participated in offline services of OMO educational platforms for older adults (e.g., tai chi, dance, or chess groups for older adults), screening questions included: “Have you attended offline activities of interest-based communities?” with age verification (>50 years). To mitigate common method bias, the study implemented a two-phase data collection procedure with temporal separation (84). The first phase assessed offline service experience, satisfaction, and demographic characteristics, yielding 814 valid responses. The second phase, conducted 24–48 h later, measured online–offline integration, perceived value, and engagement, resulting in 399 valid questionnaires. Invalid responses (e.g., identical answers or >15% missing data) were excluded, yielding an effective response rate of 39.9%. Harman’s single-factor test indicated that the first factor accounted for less than 40% of the total variance (85, 86). Additionally, a marker variable check using an irrelevant survey item (“I enjoy listening to classical music’”) revealed low correlations with core constructs (all r < 0.05, *p* > 0.05), providing further evidence that common method bias was not a major concern. We conducted a t-test using SPSS software to compare the mean values of the first 100 respondents with those of the last 100 respondents (87). The results indicated no statistically significant difference (*p* < 0.05) in the mean values between the two subgroups (first 100 respondents and last 100 respondents). The findings of this investigation confirm that nonresponse bias does not pose a significant concern. Demographic characteristics are detailed in Table 7.

**Table 7:** Demographics of respondents.

Variable	Category	Frequency	Percentage (%)
Gender	Male	191	47.87
	Female	208	52.13
Age	50–59 years	130	32.58
	60–69 years	184	46.12
	70–79 years	71	17.79
	≥80 years	14	3.51
Education	Junior high school or below	19	4.76
	High school	197	49.37
	College (Associate/Bachelor’s degree)	167	41.85
	Master’s degree or higher	16	4.01
Health	Good (physically strong, energetic, rarely requires medical consultation)	35	8.77
	Fair (moderate energy, occasionally requires medication)	224	56.14
	Chronic illness (requires long-term medication but mobile)	135	33.83
	Requiring long-term care (disabled or semi-disabled)	0	0.00
Monthly income (RMB)	<1,500	1	0.25
	1,500–3,000	40	10.03
	3,000–5,000	288	72.18
	5,000–8,000	68	17.04
	>8,000	2	0.50

### 5.3 Results

This study employed partial least squares structural equation modeling (PLS-SEM) for data analysis. The selection of PLS-SEM was justified by two primary reasons: First, PLS-SEM accommodates non-normally distributed data, delivering robust results even under high skewness conditions (88). Second, PLS-SEM excels in analyzing complex models involving multiple variables (89).

#### 5.3.1 Measurement model

Reliability was assessed using Cronbach’s *α* coefficients, and as presented in Table 8, all constructs exhibited α values exceeding the 0.7 threshold, confirming high internal consistency. Convergent validity was evaluated against three criteria: factor loadings >0.60, average variance extracted (AVE) > 0.50, and composite reliability (CR) > 0.70, confirming all constructs satisfied these benchmarks, establishing robust convergent validity (90). Discriminant validity was tested via the Fornell-Larcker criterion, where square roots of AVE exceeded inter-construct correlations (Table 9), and the Heterotrait-Monotrait Ratio (HTMT), with all values remaining below the 0.90 threshold (Table 10). These results verifying distinct construct dimensionality (104).

**Table 8:** Reliability and convergent validity.

Construct	Indicator	Loading	α	CR	AVE
Interaction quality	INT1	0.794	0.789	0.883	0.683
	INT2	0.830			
	INT3	0.854			
Peer-to-peer interaction quality	PTP1	0.856	0.848	0.856	0.767
	PTP2	0.906			
	PTP3	0.864			
Satisfaction with offline service	SAT1	0.712	0.810	0.875	0.638
	SAT2	0.749			
	SAT3	0.861			
	SAT4	0.877			
Integration of online-offline service	IOOS1	0.790	0.765	0.876	0.675
	IOOS2	0.841			
	IOOS3	0.833			
Perceived value of online service	PV1	0.935	0.834	0.876	0.750
	PV2	0.773			
	PV3	0.882			
Digital literacy	DIG1	0.733	0.749	0.841	0.569
	DIG2	0.756			
	DIG3	0.717			
	DIG4	0.809			
Engagement with online service	ENG1	0.945	0.948	0.948	0.901
	ENG2	0.953			

α, Cronbach’s alpha; CR, composite reliability; AVE, average variance extracted.

**Table 9:** Discriminant validity of constructs using Fornell-Larcker.

Variables	1	2	3	4	5	6	7	8	9	10	11	12
1. Gender												
2. Age												
3. Education												
4. Health												
5. Income												
6. Interaction quality												
7. Peer-to-peer interaction quality	−0.002^NS^	0.033 ^NS^	0.051 ^NS^	0.257^***^	0.084 ^NS^	0.516^***^	**0.876**					
8. Satisfaction with offline service	0.012 ^NS^	0.024 ^NS^	0.251^***^	0.226^***^	0.010 ^NS^	0.438^***^	0.488^***^	**0.799**				
9. Integration of online-offline service	0.006 ^NS^	0.047 ^NS^	0.126^**^	0.058^NS^	0.049 ^NS^	0.264^***^	0.250^***^	0.232^***^	**0.822**			
10. Perceived value of online service	0.027 ^NS^	0.090 ^NS^	0.165^***^	0.126^**^	0.078 ^NS^	0.344^***^	0.417^***^	0.206^**^	0.303^***^	**0.866**		
11. Digital literacy	−0.069^NS^	−0.010 ^NS^	0.040 ^NS^	0.144^**^	0.122^**^	0.087^NS^	0.072^NS^	0.052 ^NS^	0.096^NS^	0.172^***^	**0.754**	
12. Engagement with online service	0.003 ^NS^	0.078 ^NS^	−0.097 ^NS^	0.104^*^	0.108^*^	0.369^***^	0.312^***^	0.356^***^	0.198^***^	0.378^***^	0.468^***^	**0.949**
Mean	0.479	62.291	2.451	3.747	3.078	3.562	3.357	3.617	3.801	3.634	2.517	3.276
Std. deviation	0.500	8.655	0.650	0.608	0.536	0.482	0.591	0.409	0.399	0.470	0.557	0.566

The bold numbers on the diagonal are the square roots of the AVE of the variables. Gender is coded as 1 for female and 0 for male; educational attainment is coded as 1 for junior high school or below, 2 for high school, 3 for bachelor’s degree, 4 for master’s degree or above; health is coded as 1 for requiring long-term care, 2 for having a disease, 3 for average, 4 for healthy; income is coded as 1 for monthly income below 1,500 yuan, 2 for monthly income between 1,500–3,000 yuan, 3 for monthly income between 3,000–5,000 yuan, 4 for monthly income between 5,000–8,000 yuan, 5 for monthly income above 8,000 yuan. ***p = 0.001, **p = 0.01, *p = 0.05, NS, not significant (based on the two-tailed student *t*(4999) distribution).

**Table 10:** Discriminant validity of constructs using HTMTs.

Variables	1	2	3	4	5	6	7	8	9	10	11	12
1. Gender												
2. Age	0.082											
3. Education	0.017	0.197										
4. Health	0.071	0.107	0.111									
5. Income	0.008	0.027	0.115	0.163								
6. Interaction quality	0.031	0.051	0.095	0.149	0.181							
7. Peer-to-peer interaction quality	0.022	0.044	0.067	0.278	0.308	0.620						
8. Satisfaction with offline service	0.064	0.027	0.277	0.253	0.234	0.487	0.571					
9. Integration of online-offline service	0.053	0.060	0.147	0.062	0.081	0.306	0.295	0.291				
10. Perceived value of online service	0.028	0.011	0.179	0.138	0.092	0.422	0.489	0.225	0.357			
11. Digital literacy	0.087	0.042	0.055	0.176	0.148	0.363	0.349	0.185	0.235	0.340		
12. Engagement with online service	0.003	0.081	0.100	0.118	0.050	0.433	0.357	0.411	0.226	0.435	0.559	

Prior to hypothesis testing, multicollinearity in both measurement and structural models was assessed via variance inflation factors (VIF). Per academic consensus, VIF values below 10 indicate no severe multicollinearity. As presented in Tables 11, 12, all variables exhibited VIF values ranging between 1.21 and 7.83—well below the threshold of 10—confirming the absence of significant multicollinearity (104).

**Table 11:** VIF values for the measurement model.

Variable	Items	VIF
Interaction quality	INT1	2.574
	INT2	2.756
	INT3	1.297
Peer-to-peer interaction quality	PTP1	2.122
	PTP2	2.883
	PTP3	1.921
Satisfaction with offline service	SAT1	1.437
	SAT2	1.383
	SAT3	2.494
	SAT4	2.588
Integration of online-offline service	IOOS1	1.667
	IOOS2	1.447
	IOOS3	1.614
Perceived value of online service	PV1	2.907
	PV2	1.705
	PV3	2.244
Digital literacy	DIG1	1.872
	DIG2	2.359
	DIG3	2.434
	DIG4	3.487
Engagement with online service	ENG1	2.811
	ENG2	2.812

**Table 12:** VIF values for the structural model.

Variables	1	2	3	4	5	6	7	8	9	10	11	12
1. Gender												1.021
2. Age												1.095
3. Education												1.078
4. Health												1.697
5. Income												1.683
6. Interaction quality									1.363			
7. Peer-to-peer interaction quality									1.363			
8. Satisfaction with offline service										1.057		
9. Integration of online-offline service										1.057		
10. Perceived value of online service												1.171
11. Digital literacy												1.125
12. Engagement with online service												

#### 5.3.2 Structural model

##### 5.3.2.1 Path coefficient test

The sign and significance of the path coefficient (*β*) are used as test indicators to check whether the hypothesis is supported. The sign of the path coefficient should be consistent with the relevant hypothesis and be significant at the 95% confidence level.

As shown in Table 13, the offline service experience (interaction quality, peer-to-peer interaction quality) is significantly positively correlated with satisfaction with offline service (*β* = 0.251, *p* < 0.001; *β* = 0.362, *p* < 0.001), thus supporting H1 and H2. Satisfaction with offline service has a significantly positive correlation with the perceived value of online service (*β* = 0.144, *p* < 0.001), thus supporting H3. In addition, the integration degree of offline and online service has a significantly positive correlation with the perceived value of online service by older adults (*β* = 0.276, *p* < 0.001), so H4 is supported. The perceived value of online service by older adults is significantly positively correlated with their intention to engage with online service (*β* = 0.286, *p* < 0.001), thus supporting H5. Digital literacy is significantly positively correlated with older adults’s engagement with online service (*β* = 0.392, *p* < 0.001), so H6 is supported. The interaction term between digital literacy and the perceived value of online service is significantly positively correlated with older adults’ engagement with online service (*β* = 0.288, *p* < 0.001), thus supporting H7.

**Table 13:** Hypothesis testing results.

Path	*β*	*t*-values	Conclusion
Gender → Engagement with online service	0.009	0.284^NS^	
Age → Engagement with online service	0.041	1.071^NS^	
Education → Engagement with online service	0.076	0.507^NS^	
Health → Engagement with online service	0.060	1.120^NS^	
Income → Engagement with online service	0.046	1.002^NS^	
*H1*: Interaction quality → Satisfaction with offline service	0.251	5.308***	Supported
*H2*: Peer-to-peer interaction quality → Satisfaction with offline service	0.362	7.526***	Supported
*H3*: Satisfaction with offline service → Perceived value of online service	0.144	2.813***	Supported
*H4*: Integration of online-offline service → Perceived value of online service	0.276	6.008***	Supported
*H5*: Perceived value of online service → Engagement with online service	0.286	7.429***	Supported
*H6*: Digital literacy → Engagement with online service	0.392	9.485***	Supported
*H7*: Digital literacy × Perceived value of online service → Engagement with online service	0.288	6.251***	Supported

Gender is coded as 1 for female and 0 for male; educational attainment is coded as 1 for junior high school or below, 2 for high school, 3 for bachelor’s degree, 4 for master’s degree or above; health is coded as 1 for requiring long-term care, 2 for having a disease, 3 for average, 4 for healthy; income is coded as 1 for a monthly income of less than 1,500 yuan, 2 for a monthly income between 1,500–3,000 yuan, 3 for a monthly income between 3,000–5,000 yuan, 4 for a monthly income between 5,000–8,000 yuan, 5 for a monthly income of more than 8,000 yuan. ***p = 0.001, **p = 0.01, *p = 0.05, NS, not significant (based on the two-tailed Student’s *t*(4999) distribution).

**Figure 3:**
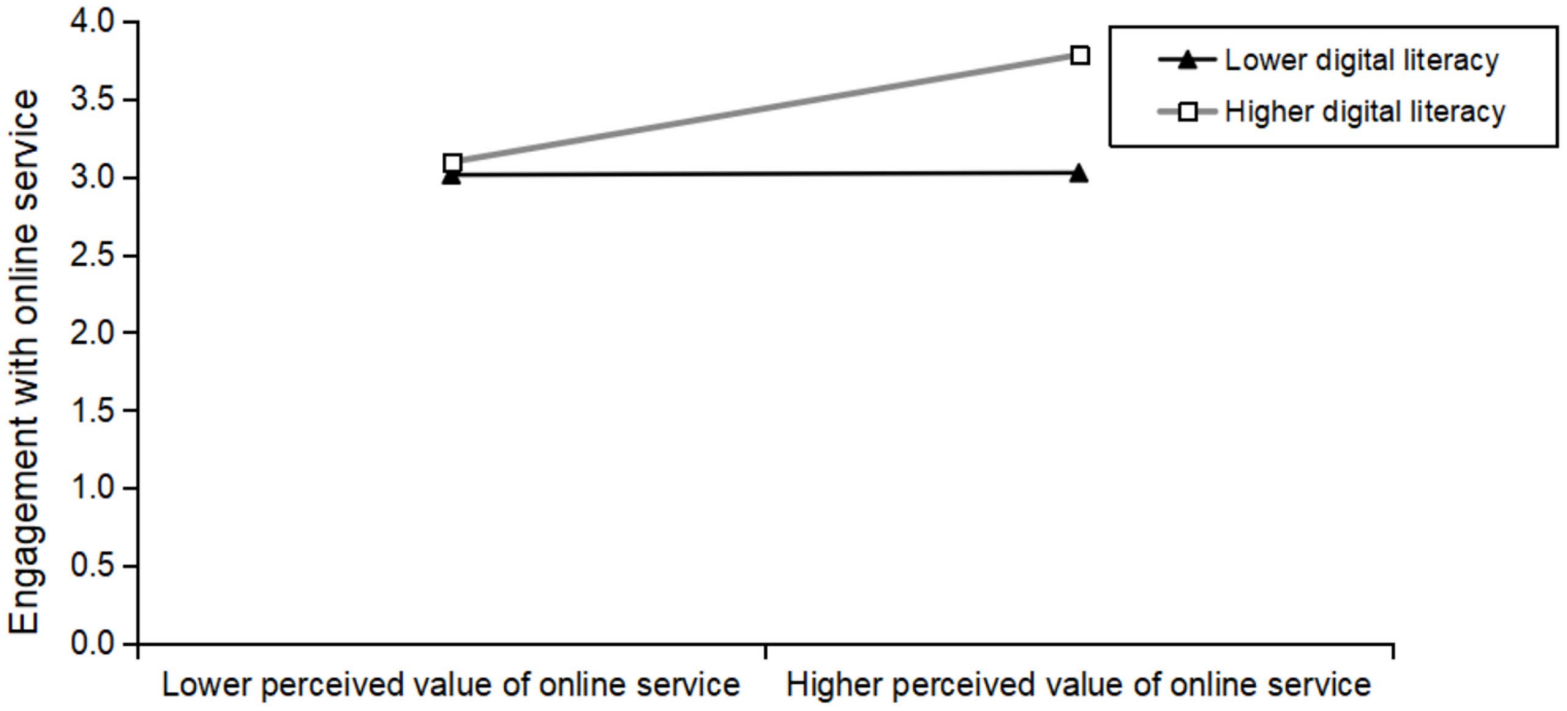
Simple slope plot of the moderating effect of digital literacy.

The study further used simple slope analysis (109) to more deeply analyze the moderating effect of digital literacy on the perceived value of online service and engagement with online service. The research results show that: under the low level of digital literacy (one standard deviation below the average), the impact of the perceived value of online service on engagement with online service is not significant (simple slope = 0.014, *p* > 0.05). Under the high level of digital literacy (one standard deviation above the average), the perceived value of online service has a significant positive impact on engagement with online service (simple slope = 0.733, *p* < 0.001). Figure 3 is the corresponding simple slope analysis chart.

##### 5.3.2.2 Model robustness test

The research further evaluates the robustness of the model. Stone-Geisser’s Q^2^ is used to test the predictive relevance of the model. As shown in Table 14, the Stone-Geisser’s Q^2^ values of satisfaction with offline service, online service perceived value, and engagement with online service are 0.175, 0.077, and 0.324 respectively, all greater than the threshold of 0, indicating that the model has a certain predictive relevance. The goodness of fit (GoF) value of the model is 0.632, indicating that the goodness of fit of the model is relatively large (110−112). The explained variance (R^2^) of the endogenous structure is used to test the degree to which the dependent variable explains the independent variable. The R^2^ values of satisfaction with offline service, online service perceived value, and engagement with online service are 0.286, 0.111, and 0.374 respectively, which are greater than the threshold of 0.1 (64). In summary, the structural model of this study is relatively robust.

**Table 14:** Model robustness test (Q^2^ values).

Variables	SSO	SSE	Q^2^ (=1-SSE/SSO)
1. Gender	399.000	399.000	
2. Age	399.000	399.000	
3. Education	399.000	399.000	
4. Health	399.000	399.000	
5. Income	399.000	399.000	
6. Interaction quality	1197.000	1197.000	
7. Peer-to-peer interaction quality	1197.000	1197.000	
8. Satisfaction with offline service	1596.000	1317.593	0.175
9. Integration of online-offline service	1197.000	1197.000	
10. Perceived value of online service	1197.000	1104.756	0.077
11. Digital literacy	1596.000	1596.000	
12. Engagement with online service	798.00	539.779	0.324

## 6 Discussion and implications

### 6.1 Conclusion

This study investigates the transition process of older adults from offline to online service within OMO contexts, employing a mixed-methods approach integrating qualitative and empirical research. The study examines how offline service experiences influence older adults’ engagement with online service. Key findings demonstrate that: Offline service experiences positively influence satisfaction with offline service; Offline satisfaction and integration of online-offline service jointly enhance perceived value of online service; Perceived value subsequently drives engagement with online service; Digital literacy directly strengthens older adults’s engagement with online service while moderating the relationship between elderly’s perceived value and engagement with online service. Collectively, these results validate the proposed theoretical model in explaining older adults’ transition from offline to engagement with online service.

This study reveals that within offline service experiences, perceived quality of interactions with service providers and peer-to-peer interaction quality are positively correlated with older adults’ satisfaction with offline service. While prior research has established the impact of interaction quality of provider-customer on satisfaction (91), the influence of non-provider actors—particularly peer-to-peer interaction quality—remains understudied. Our findings demonstrate that interactions with both providers and elderly fellow significantly shape older adults’ satisfaction with offline service. Crucially, older adults’ offline service experiences constitute not only a process of receiving provider-delivered services but also a dimension of social engagement with peers sharing common interests.

Additionally, this study identifies a significant positive correlation between older adults’ satisfaction with offline service and their perceived value of online service. This finding contrasts sharply with prior e-commerce research on service channel migration (65), where online and offline service typically exhibit a substitutive relationship (45, 72). In contrast, educational platforms targeting older adults demonstrate an integrated relationship between offline and online service. Older adult customers engage with online service to access information complementary to offline experiences, expand communication channels with service providers and peers as well as to obtain comprehensive service experiences (65). Thus, higher offline satisfaction corresponds to stronger perceived value of online service. Furthermore, greater integration of online-offline service predicts stronger willingness to engage with online service. Additionally, this study reveals that older adults’ digital literacy exhibits: A positive correlation with engagement with online service; A positive moderating effect on the relationship between perceived value of online service and older adults online engagement. While prior research has established the direct link between digital literacy and online engagement (70), its moderating role remains underexplored. This study pioneers the identification of digital literacy as a significant moderator behavioral drivers and elderly engagement with online service.

### 6.2 Theoretical contributions

This study develops a grounded theory–based model describing how older adults transition from offline to digital engagement in OMO contexts. Positive offline experiences enhance offline satisfaction and, together with online-offline integration, increase the perceived value of online services, which subsequently promotes engagement. Rather than diverging from prior work, our model complements youth-focused OMO studies (92, 93, 101) by providing an age-specific perspective. Our finding that offline service quality shapes engagement aligns with and elaborates Sun et al.’s (4) behavioral design perspective, particularly their call for anxiety-reducing pathways that support older adults’ digital well-being through stronger social integration. The limited uptake of socially disconnected online platforms further highlights the role of offline providers as facilitators of digital participation, enriching discussions that have typically emphasized online-driven mechanisms.

This study reveals that older adults’ experiences with offline services positively correlate with online engagement. Our result that offline peer interaction catalyzes sustained online participation complements Bartomeu et al. (50), substantiating that tai chi/calligraphy sessions provide the relational foundation for older adults’ engagement in online communities. This finding contrasts with service channel migration literature (38, 92) that assumes substitutive relationships. OMO platforms operate through content integration, personnel continuity, and data convergence (43), demonstrating that positive offline experiences drive online engagement for older adults, enriching channel migration literature with an older adult–specific framework.

The study further identifies that digital literacy directly enhances and moderates older adults’ engagement with online services. Our model conceptualizes OMO platforms as mechanisms for community-based digital backfeeding, offering feasible strategies to reduce literacy gaps while generating mutual benefit. Whereas previous study highlight family-based digital support through WhatsApp (94, 95) (103, , 104, 105, 106, 107, 108, 109, 110, 111, 112), our findings complement this literature by examining peer-learning dynamics within China’s OMO communities, clarifying boundary conditions for adoption and contributing to gerontological research on technology acceptance.

### 6.3 Practical implications

First, findings show that strong offline services support social inclusion and psychological well-being. Simple activities—such as square dancing or community workshops—matter a lot. When these activities highlight peer support and warm interactions, older adults feel less lonely and less depressed. They also gain more chances to make friends and try new lifestyles. These everyday interactions help build community connections and strengthen social ties. For this reason, staff members are not expected to only “run” activities. They also play a key role in building trust, encouraging mutual help, and growing social capital.

Second, our results show that online–offline integration increases perceived value in clear ways. Older adults prefer platforms where online content matches what they see and feel offline. If the content is fragmented, their engagement drops. OMO systems work best when they operate as one coherent learning environment. In this model, offline workshops can continue naturally online. This consistent experience supports long-term behavior change. It also helps older adults maintain social support in their daily lives.

Third, digital literacy drives engagement and shapes how older adults use OMO services. This creates an important equity issue. Digitally skilled seniors can act as peer mentors, but relying only on them may worsen disparities. Many older adults still face digital barriers. Public health practice should therefore support adaptive design. This includes simpler interfaces and intergenerational training activities. These approaches help low-literacy users participate more confidently. When such practices are embedded into OMO systems, the platforms can serve a wider and more diverse group of older adults. They can also reduce digital gaps and make learning resources easier to access.

### 6.4 Limitations and future work

Although this study yields many valuable findings and implications, it is still preliminary and has several limitations. Firstly, due to the purpose and nature of the study, the questionnaires were completed by the same person during the survey process. Although we have carried out some control and post-testing for common method variance, it cannot be completely eliminated. Further research could collect data from multiple sources. Secondly, this study might not cover all important variables. Further research can take into account other variables, such as organizational performance indicators, to offer managers more practical implications. Fourth, snowball and self-reported sampling limit generalizability, potentially overrepresenting digitally engaged older adults in urban China. Future research should employ stratified sampling across regions and cultures to examine how contextual factors influence engagement behaviors. Fifth, despite the inclusion of control variables – a standard approach in the literature to address endogeneity in structural equation modeling – completely mitigating endogeneity remains difficult. Further research should attempt to incorporate experimental methodologies to strengthen the robustness of our conclusions. Moreover, the scope of this study is constrained by its contextual and demographic focus. Our findings are rooted in the Chinese OMO model, which features unique institutional and adoption patterns, primarily catering to the ‘young-old’ (or ‘new-old’) demographic. As a result, the sample reflects this concentration of users, and the conclusions may not be easily generalized to other cultural contexts or to older populations with different age distributions. Future research should broaden its scope to include cross-national comparisons and encompass a wider range of older adults, in order to assess the generalizability and limitations of the current findings.

## Data Availability

The raw data supporting the conclusions of this article will be made available by the authors, without undue reservation.

## Footnotes

1
https://www.credamo.com
